# The Public Health Impact of a Publically Available, Environmental Database of Microbial Genomes

**DOI:** 10.3389/fmicb.2017.00808

**Published:** 2017-05-09

**Authors:** Eric L. Stevens, Ruth Timme, Eric W. Brown, Marc W. Allard, Errol Strain, Kelly Bunning, Steven Musser

**Affiliations:** Center for Food Safety and Applied Nutrition, U.S. Food and Drug AdministrationCollege Park, MD, USA

**Keywords:** whole-genome sequencing, databases, genetic, foodborne pathogens, foodborne illness prevention, public health

Imagine a public health resource that contains the whole-genomic sequences of tens of thousands of microbial pathogens that can be accessed by anyone in the world at any time and without cost or registration. Furthermore, each of these stored genetic sequences also has a wealth of metadata attached to it that provides additional and useful identifying information. From providing the geographic location of where the food or environmental isolate was collected to identifying the year that the isolate was obtained, this combination of genomic information and its accompanying and descriptive information (i.e., metadata) can be used to inform the interpretation of phylogenetic trees constructed using the sequence data. Finally, now imagine that this process is automated and each day the phylogenetic tree of a specific species is updated, allowing public health scientists to infer the evolutionary history and relationship of relevant isolates to not only resolve foodborne outbreaks, but to prevent them all together. One may next think to ask how far are we away from realizing this vision, and the answer is that we are already there.

The underlying science behind using whole-genome sequencing (WGS) data to link clinical isolates back to its environmental or contaminated food source is simple: is the DNA of the environmental or food isolate and the clinical isolate genetically related to each other? That is, are the 3–5 million nucleotides that make up the whole-genomic sequences of those isolates under consideration identical or do they differ by only a few nucleotide changes [e.g., A to G mutation; called a single nucleotide polymorphism (SNP)]. Since foodborne pathogens often have short generation times under optimal growing conditions, it is expected that a small number of mutations, or SNPs, will be acquired from the time of product contamination to the isolation of the clinical specimen. Therefore, there could be a range as to the number of SNP differences seen among clinical, food, and environmental isolates that are all part of an outbreak, but these can still easily cluster together and even be distinct from non-outbreak related isolates. That is the power and utility of using genomic sequence data.

However, using genetic information for identification purposes is not a novel concept. In fact, much of how bacterial DNA is currently being used across various food safety applications is based on similar methods that have been employed using human or viral genetic information. For instance, comparing the DNA sample collected at a crime scene and comparing that to the DNA of multiple suspects is very similar to comparing a bacterial isolate derived from a clinical patient and comparing that to different environmental or food isolates that could have led to the cause of the clinical illness. Matching of human DNA has also been used to determine the paternity of a child for where there is more than one potential father, which is also very similar to determining the source of an environmental isolate from a production facility to one of several possible ingredients that could have introduced the contamination into the facility from an earlier part in the supply chain. This approach has also been applied to delimit both the transmission and spread of viral outbreaks, including HIV (Ou et al., 1992), Ebola (Holmes et al., 2016), and Cholera (Chin et al., 2011), much in the same way that it is currently being used as a real-time molecular epidemiological tool for foodborne disease surveillance. Furthermore, WGS has also been used to track antimicrobial-resistant bacterial infections in real-time in a hospital setting (Snitkin et al., 2012).

Perhaps one of the most promising applications of building a microbial reference database filled with the genomic sequences of environmental and food isolates collected from regions all around the world is almost identical to how human DNA sequences can reveal from which countries a person's ancestors most likely came from. To think that combining microbial DNA information and its geographic source can speed up outbreak investigations by suggesting potential sources is just one of the many public health benefits of utilizing this advancing technology. This application and use is more than just promising: it is essential considering the increasingly global food supply and its resulting supply chain that feeds both domestic and international populations. Indeed, the application of this technology for foodborne outbreak investigations has already begun to reduce the time required to identify the source of the outbreak. By narrowing the epidemiological focus using WGS' increased resolution over traditional subtyping methods (e.g., PFGE) in differentiating between outbreak and non-outbreak related samples, less time is spent tracking the outbreak to its source in order to remove it from the food supply.

However, this technology also has the potential to severely limit or prevent outbreaks from occurring when used as part of preventative controls, which is the focus of the recent Food Safety and Modernization Act (FSMA; Pub. L. 111–353). Routine environmental and product monitoring by both industry and regulatory agencies has already been used to identify and link a contaminated product to a clinical illness very early on during the course of an outbreak, allowing for a quick response that led to a recall of smaller amounts of the contaminated product. While no one can be certain, it is likely that these actions prevented many other individuals from becoming sick and likely saved the company more than if a greater amount of the contaminated product had made its way to market. Decreasing the amount of recalled product, as well as reducing the number of sick individuals saves money both in terms of fewer lawsuits and protecting brand recognition.

Further, uses of this technology within production facilities have demonstrated its ability to resolve between transient and resident pathogens, giving industry a powerful, and precise tool to track and trace sources of contamination by themselves as part of their requirements for environmental monitoring under FSMA. Companies are already routinely employing WGS to resolve contaminating niche locations within their production lines, preventing their finished products from ever becoming contaminated. It is this use of WGS that will perhaps have the biggest impact on public health in the future by significantly reducing the number of contaminated products entering the market, thereby decreasing both the frequency and size of foodborne outbreaks by making the food supply safer.

Regardless of whether WGS is used within a preventative control or outbreak response framework, its success is predicated on two things: (1) using the genomic sequences of isolates to differentiate between related and unrelated samples; and (2) having a sufficient number of reference isolates from which to compare against. For preventative controls, only the isolates specific to that supply chain (e.g., environmental sampling or isolates from ingredients coming in or out of the production facility) are necessary, and these databases could be private or industry-specific. However, if outbreak detection or tracking isolates across the global supply chain is the focus of WGS technology, then it is paramount to have a freely-available database filled with environmental and food isolates from all over the world that anyone can access.

And that is precisely what the United States Food and Drug Administration (FDA) realized at the same time that the cost of WGS began to rapidly decrease in the late 2000s. Therefore, in 2011 FDA launched GenomeTrakr (FDA, 2016), a genomic repository for storing the sequences of food or environmental isolates and housed at the National Center for Biotechnology Information (NCBI, 2016). This distributed network of federal, state, international, and public health laboratories houses mainly food and environmental isolates and combines this genomic information with an isolate's descriptive metadata. As previously described, this combination serves as an important resource for both public health agencies and industry to compare the genetic sequences of both clinical and environmental isolates against.

GenomeTrakr currently contains this information for more than 70,000 isolates, and continues to grow at a remarkable pace as more states and countries understand the benefit of WGS technology for food safety (see Figure 1). With new laboratories and partners joining the network each month, GenomeTrakr's expanding reference library is more likely to house a sequenced isolate that is genetically related to a genome that is queried against those 70,000 isolates. This ability to provide for a possible genetic match only becomes more probable as environmental and product sampling increases across the numerous production regions around the world. In other words, the geographic information attached to each isolate is then better able to supply critical information helpful in resolving the myriad possible sources of contamination if many isolates are being sampled from a uniform distribution (i.e., isolates come from many states, countries, and from a wide array of food commodities that represent variation seen among the different bacterial species).

**Figure 1:**
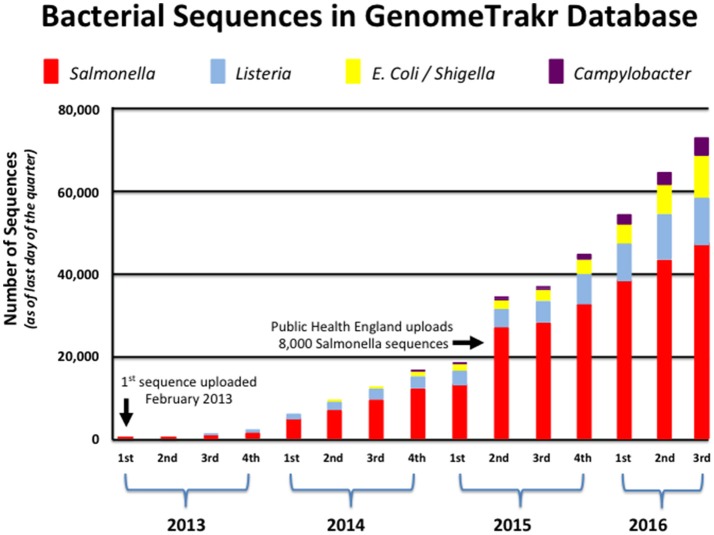
**Growth of bacterial genomes within GenomeTrakr (2013–2016)**. This figure shows the number of bacterial genomes that have been added to FDA's GenomeTrakr at the end of each quarter spanning 2013–2016. Four pathogens (*Salmonella, Listeria, E. coli*/*Shigella*, and *Campylobacter*) are the dominant foodborne pathogens collected and made available to the public.

It is inevitable that the number of sequenced environmental and food isolates will only continue to increase as more and more countries and industries embrace WGS technology its position as a single microbiological test (e.g., serotyping, antimicrobial resistance, pathogenicity, etc.). These genomic sequences—and the associated metadata—need to be made available for its successful use as a public health resource. However, there are serious concerns that arise from the sharing of such potentially sensitive data, especially when it could be used to self-implicate industry if supplied environmental or product samples match to clinical isolates. On the other hand, routine use of this technology by industry within their own production lines and supply chains can help mitigate that problem by becoming aware of and then correcting the contamination internally before it is able to result in a clinical illness. Industry could also use this technology to identify possible sources of contamination within their supply chain, especially if they receive raw ingredients from multiple and distinct sources. There are also concerns that this technology could adversely affect developing or transitional countries, and even smaller companies that lack the necessary resources able to robustly utilize this technology for their own purposes of surveillance and preventative controls. It is hoped that all stakeholders will continue to engage in meaningful dialogue to ensure the most beneficial use of this technology for public health while balancing private and commercial interests.

Nevertheless, environmental sampling and the availability of an isolate's genomic information is fundamental in order to gain an understanding into the inherent geographic variation and spread of potential foodborne pathogens coming out of the fields and at various points along the supply chain. The public health benefit of providing public access to this data will only become more vital as our ability to process and analyze this data become more sophisticated. Indeed, GenomeTrakr has already successfully demonstrated how a large database of genomic sequences and metadata can be used effectively for food safety within the United States by not only helping to decrease the length of foodborne outbreak investigations but by detecting outbreaks earlier. Going forward, GenomeTrakr can serve as a model for a large database of environmental isolates from all over the world that can now be utilized for global public health benefit, and it is imagined that this resource will be beneficial to both public health agencies and industry.

## Author contributions

Wrote the paper: ES, RT, MA, ES, KB, SM, and EB.

## Funding

This work was supported by the Center for Food Safety and Applied Nutrition at the U.S. Food and Drug Administration.

### Conflict of interest statement

The authors declare that the research was conducted in the absence of any commercial or financial relationships that could be construed as a potential conflict of interest.
